# Blood clots affect the response of tympanic membrane perforations to gelfoam grafting after ventilation tube insertion

**DOI:** 10.1186/s40463-021-00496-z

**Published:** 2021-01-28

**Authors:** Zhengcai Lou

**Affiliations:** Department of Otorhinolaryngology, Yiwu central Hospital, 699 Jiangdong Road, Yiwu city, 322000 Zhejiang Province China

**Keywords:** Myringoplasty, Ventilation tube insertion, Tympanic membrane perforation, Gelfoam graft

## Abstract

Chronic tympanic membrane (TM) perforation associated with ventilation tube (VT) insertion was commonly encountered in pediatric patients with chronic otitis media with effusion (COME) treatment and eustachian tube dysfunction. The persistent perforation require surgical closure by myringoplasty. Song JS et al. recently a paper entitled: “Evaluating short and long term outcomes following pediatric myringoplasty with gelfoam graft for tympanic membrane perforation following ventilation tube insertion.” In their study, the authors performed gelfoam myringoplasty to repair the perforations following VT insertion in children and compare the successful TM closure rate among different graft materials. The authors believed that gelfoam alone was superior to hyaluronic acid (HA), tragal cartilage (TC), and gelfoam-plus-temporal fascia (TF). The sample size is unbalanced and incommensurable between gelfoam and other graft materials. In addition, a confounding factor was added in the gelfoam group, thereby affected the assessment of TM closure. Thus, the conclusion is not rigorous and scientific.

Chronic tympanic membrane (TM) perforation is a common complication of chronic otitis media with effusion (COME) and eustachian tube dysfunction that develops after ventilation tube (VT) insertion in children. The persistent perforation require surgical closure by myringoplasty. Several graft materials have been evaluated in an effort to optimize successful outcomes following myringoplasty, including hyaluronic acid (HA), autologous fat grafts, tragal cartilage (TC), gelfoam and temporal fascia.

(TF). Song et al. [1] recently published a paper entitled: “Evaluating short and long term outcomes following pediatric myringoplasty with gelfoam graft for tympanic membrane perforation following ventilation tube insertion.” The cited authors performed gelfoam myringoplasty to treat TMPs following VT insertion. In this technique, small amount of blood was absorbed into the gelfoam to create a little ‘blood patch’. In addition, the authors compared the success TM closure rate between the gelfoam and other graft materials. The authors believed that pediatric myringoplasty with gelfoam graft material is a safe and viable alternative with favorable short and long term clinical and audiometric outcomes, gelfoam alone was superior to HA, TC, and TF. However, I just want to declare some points that limit the power of this study.

The authors reported that, at initial follow-up, 90.6% of the gelfoam (GF) group exhibited successful TM closure, compared to 50% of the HA, 85.7% of the TC, and 66.7% of the TF groups in this study [1]. The gelfoam alone was superior to HA, TC, and TF. The sample size is 85 patients in GF, 16 patients in HA, 7 patients in TC, and 6 patients in TF groups. The sample size is unbalanced and incommensurable between gelfoam and other graft materials. We presume that the results of Song et al. may be attributable to the smaller numbers of patients in the HA, TC, and TF groups, compared to the GF group. The TM closure shoud be matched by increasing the sample numbers of other graft materials. In addition, most TM perforations that develop after prolonged VT insertion are small; thus, they are similar to chronic traumatic TM perforations, but not to the TM Perforations associated with COME. Niklasson et al. [2] used gelfoam plugs to repair small, chronic TM perforations; the closure rate was 83%.

The authors described “Once the tube is removed or the perforation edge is refreshed, then the gelfoam is flattened and cut into multiple smaller square pieces of variable size to match the perforation, and overlaid onto the perforation to completely cover the perforation edge. Suction is not generally used as the small amount of blood from the refreshed edges absorb into the gelfoam to create a little ‘blood patch’. In the absence of bleeding, the adjacent area of the tympanic membrane is scratched superficially with the Schuknecht needle to entice a small droplet of blood prior to placing the gelfoam. The gelfoam is not allowed to straddle the hole. No drops are used to prolong patch placement during self-dissolution. Patients are asked to keep the ear dry for 4 weeks.” [1]. The blood clots play important roles in terms of eardrum healing. Soaking of gelfoam in blood increases the duration of perforation moistness and blood component lifetimes, thereby facilitating healing. The serosanguinous discharge from a perforation accelerated eardrum healing and improved the closure rate [3]. Blood clots serve as scaffolds for epithelial migration and slowly release growth factors that stimulate eardrum healing [4]. Kakehata et al. [5] found that application of autologous serum eardrops promoted the closure of chronic TM Perforations. A few clinical studies suggested that topical application of epidermal growth factor (EGF) or basic fibroblast growth factor-2 (bFGF2) (without gelfoam patching) reset normal eardrum healing, improved the closure rate, and shortened the closure time of chronic perforations [6, 7] Lou et al. [6, 7] used topical growth factors to repair subacute perforations that had been present for more than 2 months; the closure rates were 91.7 and 96.2% when bFGF2 and EGF were applied, respectively. Lou used EGF to treat 24 patients with chronic traumatic perforations; the closure rate was 100%. Thus, the study by Song et al. [1] does not identify the key factor that improves perforation closure. It may be the blood patch, the gelfoam graft, or both in combination. Therefore, the conclusion of this study is not rigorous and scientific, further studies of large size are needed in future.

## Data Availability

The datasets supporting the conclusions are included within the article.

## Abbreviations

TM: Tympanic membrane
COME: Chronic otitis media with effusion
VT: Ventilation tube
HA: Hyaluronic acid
TC: Tragal cartilage
TF: Temporal fascia
GF: Gelfoam
EGF: Epidermal growth factor
bFGF2: Basic fibroblast growth factor-2
